# Contributions of Major Cell Populations to Sjögren’s Syndrome

**DOI:** 10.3390/jcm9093057

**Published:** 2020-09-22

**Authors:** Richard Witas, Shivai Gupta, Cuong Q. Nguyen

**Affiliations:** 1 Department of Infectious Diseases and Immunology, College of Veterinary Medicine, University of Florida, Gainesville, FL 32608, USA; rwitas@ufl.edu (R.W.); shivai.gupta@ufl.edu (S.G.); 2 Department of Oral Biology and College of Dentistry, University of Florida, Gainesville, FL 32610, USA; 3 Center of Orphaned Autoimmune Diseases, University of Florida, Gainesville, FL 32610, USA

**Keywords:** Sjögren’s syndrome, autoimmunity, salivary gland, innate cells, adaptive cells

## Abstract

Sjögren’s syndrome (SS) is a female dominated autoimmune disease characterized by lymphocytic infiltration into salivary and lacrimal glands and subsequent exocrine glandular dysfunction. SS also may exhibit a broad array of extraglandular manifestations including an elevated incidence of non-Hodgkin’s B cell lymphoma. The etiology of SS remains poorly understood, yet progress has been made in identifying progressive stages of disease using preclinical mouse models. The roles played by immune cell subtypes within these stages of disease are becoming increasingly well understood, though significant gaps in knowledge still remain. There is evidence for distinct involvement from both innate and adaptive immune cells, where cells of the innate immune system establish a proinflammatory environment characterized by a type I interferon (IFN) signature that facilitates propagation of the disease by further activating T and B cell subsets to generate autoantibodies and participate in glandular destruction. This review will discuss the evidence for participation in disease pathogenesis by various classes of immune cells and glandular epithelial cells based upon data from both preclinical mouse models and human patients. Further examination of the contributions of glandular and immune cell subtypes to SS will be necessary to identify additional therapeutic targets that may lead to better management of the disease.

## 1. Introduction

Sjögren’s syndrome (SS) is the second most common autoimmune disorder after rheumatoid arthritis (RA) [1]. Like systemic lupus erythematosus (SLE), SS is a chronic and systemic autoimmune disease [2]. While SS is most commonly associated with xerostomia, xeropthalmia, and lymphocytic infiltration into the exocrine glands, SS patients may present with gastrointestinal symptoms, fatigue, pulmonary problems, and experience a higher incidence of non-Hodgkin’s B cell lymphoma (NHL) [2,3]. The lymphocytes that infiltrate into the exocrine glands can organize into focal structures in which germinal center-like formation is present in approximately 25% of primary SS patients [4], establishing a structure for local production of autoantibodies [4,5]. SS primarily affects women and features a highly skewed sex distribution (9:1) [6]. SS is a complex heterogenous disease that can present alone, referred to as primary SS (pSS), or as secondary SS with another autoimmune disease such as RA or SLE. While the adaptive immune cells like B and T cells have traditionally attracted the most interest due to their predominant presence in the exocrine glands and the immunological importance autoantibodies, increasing evidence shows that immune system dysfunction in SS incorporates cells of the innate immune system as well [7,8,9,10,11,12]. In this review, we aim to present the current state of knowledge on how the common cell types of the innate and adaptive immune systems contribute to SS as revealed by studies of human patients and animal models.

## 2. Disease Development

Like other autoimmune diseases, SS is considered a multifactorial disease where a susceptible genetic background requires an environmental factor trigger, such as viral infection [13], to initiate the development of disease. Genome wide association studies (GWAS) have identified several genetic risk factors for SS. Two GWAS in SS have been performed, one with patients of European descent, and another with Han Chinese patients [14,15]. Both studies identified alleles within human leukocyte antigen (HLA) Class II to be the most associated with SS, particularly alleles of the HLA-DR and HLA-DQ isotypes. While major histocompatibility complex (MHC) Class II alleles show the greatest association with SS, several non-MHC genes also possess a significant association. Many of these susceptibility genes, including *IRF5*, *STAT4*, and *IL12A* are involved in the regulation of the interferon (IFN) system [14,15]. The upregulation of IFN pathways and its stimulated genes are associated with the clinical symptoms of SS [16,17]. Over half of all pSS patients exhibit an IFN signature, and these patients typically present higher titers of anti–Sjögren’s-syndrome-related antigen A (SSA/Ro) and anti–Sjögren’s-syndrome-related antigen B (SSB/La) autoantibodies and higher disease activity as measured by the European League Against Rheumatism Disease Activity Index (ESSDAI) [18]. Additionally, an increased IFN gene signature in the salivary glands (SG) has been linked to poorer patient response to Rituximab, a chimeric mouse/human monoclonal antibody (mAb) therapy with binding specificity to CD20 [19]. Both Type I and type II IFN signatures have been detected for SS patients and genetic ablation of interferon α receptor 1 (IFNAR1), IFN-γ, or its receptor IFNγR prevent the onset of disease in the spontaneous SS models: the non-obese diabetic (NOD) mouse and it’s derivative C57BL/6.NOD-*Aec1Aec2* [20,21,22,23]. The initial events activating the IFN system remain unclear, as does how the precise nature of how the IFN signature of SS mediates disease. Type I IFNs (T1-IFN) are driven by toll-like receptor (TLR) stimulation and while capable of being produced by all nucleated cell types, they are strongly associated with cells of the innate immune system, whereas Type II IFNs are largely produced by T cells, NK cells and macrophages [24,25]. The apparent necessity of IFNs in the SS disease process together with IFN regulatory risk genes in humans, indicates a role for cells of the innate immune system as well as the adaptive in the development of disease. Indeed, therapies targeting T1-IFN and the IFN pathway continue to be investigated in SS [26].

The insidious onset of SS, coupled with generalized symptoms, overlap with other autoimmune diseases, and the complex classification/diagnostic parameters contributes partially to a frequent delay in diagnosis [2]. Due to the challenges of identifying “pre-SS patients”, understanding of the disease in humans has been limited to studying patients with advanced symptomatic disease. This deficit has contributed to a lack of understanding of pathological events preceding observable symptoms. Therefore, in an attempt to elucidate the early patho-immunological processes, many induced and spontaneous mouse models for SS have been developed and used to study disease progression [27]. These mouse models can differ greatly in their SS disease manifestations. For example, NOD mice develop well characterized salivary gland pathology with less lacrimal gland (LG) involvement, whereas thrombospondin-1 (TSP-1) deficient mice experience more severe LG disease [28]. These discrepancies between models mirror the heterogenous presentation of SS in human patients. Critically, the disease profile of individual mouse models can mimic that of subgroups of patients (IFN^+^, IFN^−^, etc.) thereby facilitating understanding of disease in these subgroups. Much of the work on spontaneous models has been done using the NOD mouse and its derivatives [29]. In studying SS progression in C57BL/6.NOD-*Aec1Aec2* mice, we were able to identify 3 distinct but overlapping phases of disease [27]. C57BL/6.NOD-*Aec1Aec2* mice develop SS symptoms temporally and phenotypically similar to NOD mice but without the presence of diabetes, making them an ideal candidate to study spontaneous pSS [30,31,32]. Phase 1 (0–8 weeks) is characterized by acinar epithelial cell death and delayed salivary gland (SG) morphogenesis. Phase 2 (8–16 weeks), where IFN stimulated genes become activated, coincides with migration of macrophages and dendritic cells (DCs), followed by CD4^+^T and B220^+^B lymphocytes, and the emergence of autoantibodies. Finally, in Phase 3 (16 weeks onward) there is overt clinical disease where a progressive measurable loss of exocrine gland function occurs. Disease development in C57BL/6.NOD-*Aec1Aec2* mice shares some similarities to other SS mouse models, even if different glands are targeted. For example, increased apoptosis was observed at 8 weeks in the LGs of TSP-1-deficient mice, which can also be seen in the SG of C57BL/6.NOD-*Aec1Aec2* mice. Additionally, TSP-1 mice displayed ocular surface damage at 12 weeks with an increase in SSA/Ro and SSB/La antibodies detected at 12–16 weeks. Finally, infiltrates primarily composed of CD4^+^ T cells were discovered in the LGs of TSP-1-deficient mice with increased expression of Th1 and Th17 and related transcription factors. Similar observation was seen in the SG of C57BL/6.NOD-*Aec1Aec2* mice at similar age [28]. Together these findings offer insights as to how the aberrant activity of both innate and adaptive immune cells mediate the pathogenesis of SS. 

## 3. Innate Immune Cells

### 3.1. Dendritic Cells

Considering that SS is characterized by overstimulation of the immune system and IFN signature, the role of DCs in SS has been the subject of considerable study [33]. DCs can be subdivided into three main types: conventional or myeloid DCs (mDCs), which are the most potent antigen presenting cells (APCs) of the immune system; plasmacytoid DCs (pDCs), the foremost IFN-α producing cell; and follicular dendritic cells (fDCs), which are not from the hematopoietic lineage and are critically involved in B cell development in germinal centers (GCs) [34,35]. DCs are one of the first cell types to infiltrate the minor SG of patients and submandibular glands of NOD mice [36,37]. Their prevalence is negatively correlated with lesion severity, whereas fDC frequency is unaffected by lesion severity. fDCs are organized into networks within GCs in severe lesions within the glands [38]. 

mDCs are cells of hematopoietic origin and include a number of tissue specific subtypes, such as Langerhan’s cells. Immature DCs disseminate through the blood to inhabit peripheral tissues where they sample the local environment through endocytosis. DCs that have encountered antigen migrate to the secondary lymphoid tissue and develop into mature DCs [39]. They are unique in their capacity to both prime T cells and participate in peripheral tolerance [40]. Immature DCs have reduced frequency in primary and secondary SS patient blood, while mature DCs are found at increased frequency within the SGs [41,42]. DCs isolated from NOD mouse SGs lacked the expression of the regulatory chemokine receptor CCR5. The absence of CCR5 on DCs contributes to an increased expression of the T helper (Th) 1 cytokine IL-12, thereby enhancing the activity of the adaptive cellular response through Th1 cells and establishing a more proinflammatory environment [43]. Patient monocyte derived DCs (moDCs) were reported to express increased HLA-DR compared control moDCs, suggesting more antigen presenting activity. moDCs from SS patients secreted higher levels of IL-12p40 than moDCs from control patients both upon TLR7/8 stimulation [44] and under basal conditions [45]. 

pDCs are a rare subset of DC best known for their production of T1-IFN upon stimulation of TLR 7 and 9 [46]. pSS patients exhibit low levels of serum T1-IFN but have elevated levels in the minor SG (mSG) [47], and reduced circulating pDC within peripheral blood [48]. However, while diminished in number, pDCs of pSS patients present in peripheral blood expressed high levels of CD40 and CD86 [49]. Microarray analysis of mSGs biopsies showed significant activation of both T1 and T2-IFN pathways with elevated numbers of pDCs [50]. Analysis of pSS pDCS has revealed dysregulated miRNAs relating to apoptosis, antigen presentation, and cytokine production [51]. Furthermore, pDCs from pSS patients demonstrated increased pro-inflammatory cytokine production [52]. Finally, it has been suggested that pDC recognition of apoptotic cell debris drives the loss of immune tolerance in SS [7].

fDCs are stromal cells within GCs that lack MHC-II expression and instead present antigen-antibody complexes to B cells via complement and Fc receptors [35]. fDCs attract B cells with CXCL13 and promote positive and negative selection, isotype switching, and development of high affinity B cell receptors [53]. About 20–25% of SS patients develop ectopic GCs containing B cells, T cells, and fDC networks within the mSGs [54]. Expression of the enzyme activation-induced cytidine deaminase is critical for B cells to perform class switch recombination and somatic hypermutation, driving affinity maturation within GCs of secondary lymphoid organs. Proliferating B cells found within fDC networks in ectopic GCs express AID, indicating that these GCs are functional and a source of local antibody production and B cell expansion within the SGs of SS patients [55].

### 3.2. Macrophages

Macrophages are a broad variety of phagocytic cells of the innate immune system. Monocytes circulate within the blood and migrate to tissues where local signals can differentiate them into macrophages or DCs. While mDCs and pDCs are recognized as the master antigen presenting cells and T1-IFN producing cells respectively, macrophages also participate in both of these roles and are crucial for producing other pro-inflammatory cytokines, apoptotic corpse removal, and wound healing [56]. SS patient saliva contain high expression of the monocyte chemokine CCL2, and histological analysis of SS patient biopsies identified macrophages within infiltrates of mSGs biopsies [57,58]. Infiltrating macrophages in pSS patients were positively correlated with lesion severity [38] and IL-18, suggesting macrophage activation within the infiltrate [59]. Furthermore, high IL-18 expression by infiltrating macrophages correlated with lymphoma risk factors such as C4 hypocomplementemia and SG enlargement [59]. The polarization of macrophages remains unclear in SS. M1 polarization has been suggested to be more likely in part because proinflammatory cytokines B cell activating factor (BAFF), T1 IFN, IL-6, and IL-12, that are expressed by M1 macrophages, are detected at higher levels in SS patients [60]. Macrophage linked protease genes including cathepsins, matrix metalloproteases (MMPs), and carboxypeptidases were found to be upregulated in highly inflamed SGs biopsies from SS patients, suggesting a role for macrophages in orchestrating tissue destruction and aberrant repair processes in SS [61]. Further investigation revealed that IFN signaling drives plasmin expression by macrophages in SGs and promotes tissue destruction [62]. In addition, macrophage derived chitinases were highly expressed in mSG samples from pSS patients and were associated with disease severity [61]. While focal lymphocytic infiltration can occur in healthy individuals, negligible numbers of macrophages are observed in foci of healthy individuals [63]. 

Within the NOD mouse model for SS, macrophages have been observed to infiltrate the SGs early in disease development, and precede the arrival of B and T cells [32]. Both M1 and M2 macrophages have been observed in SGs of NOD mice [64]. Among the most important functions carried out by macrophages is the removal of the corpses of apoptotic cells, a process termed efferocytosis [65]. In the related autoimmune disease SLE, delayed removal of dead cells is believed to contribute to disease onset [66]. Delays in the uptake and disposal of dead cells can allow the corpse to progress to secondary necrosis where self-antigens can leak out and activate the immune system [67]. Considering the observation of increased apoptotic cells in the SGs of SS patients and mouse models, this concept has received some attention in SS [68,69]. Analysis of pSS patient monocytes revealed impaired phagocytosis of apoptotic cells and a corresponding defect in initiating immunosuppressive signaling in response to uptake [70]. Additional investigation into SS monocyte derived macrophages determined that SS patient macrophages suffered an intrinsic reduction in phagocytic ability, exacerbated by inhibitory IgG antibodies against apoptotic cells [71]. NOD mice, which have been employed as a model for secondary SS, display impaired efferocytosis by both bone marrow derived and peritoneal macrophages [72]. Our own observations confirm that this efferocytic defect is maintained in the SS C57BL/6.NOD-*Aec1Aec2* mouse model (unpublished data) and may be a result of defective signaling within the Tyro3, Axl, Mertk (TAM) receptor pathway [73].

### 3.3. Innate Lymphoid Cells (ILCs) and Natural Killer Cells (NK)

Innate lymphoid cells (ILCs) have received little attention in SS compared to other immune cells. However, ILCs have been observed in both human and mouse salivary gland [74,75]. Natural killer (NK) cells are ILCs that arise from the same common lymphoid progenitor as B and T cells. NK cells have well characterized roles in the elimination of tumor cells and virally infected cells [76,77]. Yet, the role of NK cells in SS remains poorly understood. NK cells have been discovered within minor SG biopsy patients of SS patients, however the cells are rarer than DCs or macrophages [38]. Despite rarity with the SS lesion, NK cells incidence was found to be positively correlated to presence of rheumatoid factor (RF) and C4 levels in the sera [38]. NKp44^+^ ILCs were found to be major producers of IL-22 in pSS patient SG, and infiltration of these cells into the SG correlated with SG inflammation [74]. Levels of NK cells in the blood of SS patients remain controversial. Szodoray et al. observed the percentage of NK cells to be increased in the peripheral blood of pSS patients [78]. In a separate study, the count of NK cells in the peripheral blood was found to be reduced in pSS patients with anti-SSA and SSB autoantibodies [79]. Greater concordance has been achieved in studies of NK activity in pSS, where NK cell activity was found to be reduced in pSS as well as SLE [80]. In addition, Izumi et al. detected decreased NK cells, decreased NK cell activity, and increased apoptotic NK cells in pSS patients [81]. Interestingly, there is evidence that NK cell contribution to SS is not through the traditionally explored lens of NK cells as killers, but rather through the regulatory capacity of NK cells [82,83]. In this study, Rusakiewicz et al. identified a new mechanism for NK involvement in SS where dysfunctional regulation by NK cells via *NCR3*/Nkp30 permits over activation of DCs, facilitating activation of lymphocytes and systemic immunity [82]. 

### 3.4. Salivary Gland Epithelial Cells (SGECs)

SGECs are one of the targets of autoimmune attack in SS as exhibited by the aberrant apoptosis that occurs in the SG. However, further scrutiny into the role of SGECs has revealed that this class of cells is not merely the bystander target, but rather an active participant in the autoimmune response [84,85]. SGECs expressed high levels of HLA-DR, costimulatory molecules CD80 and CD86, and adhesion molecules, allowing them to perform as non-professional antigen presenting cells [86]. Additionally, SGECs have been identified to be sources of multiple chemokines and proinflammatory cytokines including CXCL12, CXCL13, IL-6, IL-7, IL-22, ICOSL, and BAFF [74,87,88,89,90,91,92,93]. Local expression of certain chemokines by SGEGs, including CXCL12 and CXCL13 is believed to contribute to the formation of ectopic GCs in the glands [89]. Furthermore, the Ro52 antigen has been detected in SGECs of pSS at higher levels than control patients, and is positively associated with the severity of inflammation [94]. Enhanced endoplasmic reticulum (ER) stress detected in the SGECs of pSS patients has been hypothesized to contribute to the production of proinflammatory cytokines from SGECs [95]. Co-culture experiments discovered that SGEC expression of ICOSL and IL-6 can differentiate naïve T cells into follicular T cells, demonstrating the ability of SGECs to influence lymphocytic organization in the SGs [91]. 

TLR 1, 2, 3, 4, and 7 are known to be expressed by SGECs [96,97,98]. Furthermore, TLR3 stimulation of the SG of New Zealand Black X New Zealand White (NZB/W) F1 mice was shown to reduce salivary flow in mice [99]. Separate studies observed that TLR3 stimulation induced apoptosis in SGECs, and SGECs from pSS patients were more susceptible to anoikis induced by TLR3 stimulation [100,101]. Additionally, pSS patients were found to overexpress the costimulatory molecule B7-H3 which was determined to be able to induce apoptosis of SGECs [102]. Stimulation of patient SGECs with TLR agonists dsRNA virus and poly I:C resulted in increased BAFF expression, further demonstrating the role of SGECs as regulators of the immune response [92]. The anti-inflammatory activity of peroxisome-proliferator-activated receptor-γ (PPARγ) was found to be reduced in SS patient derived SGECs, allowing for overactive NF-κB and IL-1β pathways [103]. TLR stimulation of the SGECs, presumably from a viral infection, represents a possible initiating event in the autoimmune cascade where increased cell death, and the release of inflammatory cytokines drive an escalating cycle of inflammation [84,85,104]. Overall, like macrophages and DCs, SGECs possess the ability to both produce various chemokines, inflammatory cytokines, and act as APCs, allowing them to exert a powerful influence guiding the behavior of lymphocytes within the SGs.

## 4. Adaptive Immune Cells

### 4.1. T cells

#### 4.1.1. Th1 Cells

Th1 cells produce the inflammatory cytokines IFN-γ and TNF-*α*, and both of these cytokines regulate cell mediated immunity and activate macrophages, NK, and CD8^+^T cells [105]. SS was originally considered a Th1 dominated autoimmune disorder, but it has gradually been observed that both Th1 and Th2 cells are drivers of the disease depending on the stage. Deciphering the specific roles of the Th1 subpopulation in the disease progression in SS has been a primary goal in understanding the disease [106]. IFN-γ has a significant effect on the organ development of SGs. *Ifnγ*^−/−^ and NOD.*IfncR*^−/−^ mice have been shown to be clinically asymptomatic for SS and indicate normal acinar cell proliferation and maturation [107,108]. It has been established that IFN-γ induces expression of glandular adhesion molecules including vascular cell adhesion molecule-1 (VCAM-1), *α*4*β*1 integrin, peripheral node addressin, L-selectin and LFA-1, which facilitate the influx of inflammatory cells into glands [108,109,110]. Further transcription signature analysis of the Th1 cell type suggests that the IFN-γ regulated cytokines CCL5, CCL8, CXCL9, CXCL13, and CXCL16 (an IFN-γ regulated chemokine) attract both NK and memory T cells [109]. IFN-γ plays an important role in the perpetuation of inflammation of SS as labial salivary gland primary cell cultures from patients indicate epithelial HLA-DR expression in 80% of cultures. IFN-γ can alter tight junction function and causes an increase of permeability across the epithelium [111]. The in-vitro exposure of acinar cells to IFN-γ causes alterations in tight junction components as observed in the SGs of patients with pSS [112]. It can induce Fas mediated apoptosis in SGEC cultures, ultimately contributing to epithelial cell damage and diminished saliva secretion [113]. Other proteins like IFN inducible guanylate binding protein 1 and CD45^+^ cell infiltration are corelated and may be analyzed by the degree of CD45^+^ infiltration in the major SGs of pSS patients [107]. CCL9 and CCL19 expression is up-regulated in the salivary (SG) and LGs (LG) of NOD and C57BL/6.NOD-*Aec1Aec2* mice during disease onset, inducing other potentially disease relevant genes such as *Epsti1* and *Ubd* that show enhanced activity in the LGs of male mice [109,114]. IL-7, known to cause increased production of IFN-γ and CXCR3 via upregulation of Th1 cells, has been shown to accelerate the development of SS [115]. Okamoto et al. have shown that IκB-ζ induction is necessary for Th17 cell differentiation and is important in experimental autoimmune encephalomyelitis [116]. Similarly, Okuma et al. have determined that the STAT3-I*κ*B-*ζ* signaling pathway is essential for the development of SS-like disease, as the genetic deletion of the STAT3-I*κ*B-*ζ* signaling pathway is sufficient for the development of SS-like disease, as enhanced apoptosis is observed after deletion of the pathway in SG tissue [117]. The epithelial cell-specific STAT3-deficient mice develop SS-like inflammation with impaired IκB-ζ expression in the LGs, activating Th1 cells [117]. The disruption of STAT3-mediated IκB-ζ induction elicits the activation of self-reactive lymphocytes that causes the spontaneous development of SS. The IκB-ζ-deficient epithelial cells accelerate apoptosis even without the involvement of lymphocytes [117]. STAT3 is widely expressed in different cells and is activated by an array of cytokines and growth factors [118,119]. It controls RORγt expression and Th17 development, but alternatively it has been found that epithelial deletion of STAT3 induced SS-like symptoms. IκB-ζ expression is significantly reduced in the LGs of STAT3-deficient mice, proving that STAT3 is required for the expression of IκB-ζ [117].

IL-18, another Th1 cytokine, has been detected in CD68^+^ macrophages, ductal, and acinar cells of SGs of SS mice and is secreted at a significantly higher level in sera and the saliva of patients with SS and NOD mice [59,120,121]. It has been established that IL-18 produced by activated macrophages and T cells stimulates the inflammatory pathway within the glands [122].

#### 4.1.2. Th2 Cells

Th2 cells mediate humoral immunity and are involved in allergic immune responses in the body [123]. Th2 cells play a critical role in sustaining B cell function and conversely, B cells regulate the maintenance and expansion of both IL-4 producing cell lineages [124]. Hyperactivity of B cells, specifically overproduction of autoantibodies is observed in SS patients. This activity is attributed by cytokines secreted by Th2 cells. Th2 cells are generated following priming of CD4^+^ T cells by IL-4, resulting in the induction of the Th2 transcription factor GATA3. Th2 cells express a range of cytokines that influence B cell differentiation, eosinophil recruitment, and mucus production [125]. The signature cytokines produced by Th2 cells are IL-4, IL-5, and IL-13 but they can also produce IL-9, IL-10, IL-25, and amphiregulin [126]. Genetic ablation of IL-4 in NOD mice was able to restore normal levels of secretory function however, leukocytic infiltration and pathophysiological abnormalities in gland pathology persisted [127,128]. IL-4 has also been found to play a crucial function during the clinical manifestation of SS while having limited effect on the pathology associated with the preclinical disease. *Il4* KO mice do not produce IgG1 isotypic autoantibodies against the muscarinic acetylcholine receptor (M3R), a known autoantibody target in SS, indicating a critical role of IgG1 isotype switching in SS. Other antibodies such as IgG2a, IgG2b, IgG3, IgM, and IgA are produced against M3R [108] in both the *Il4* KO and NOD.B10-*H2^b^* mouse models. The NOD.B10-*H2^b^* mouse model has the *Stat6* gene knocked out that impairs the capability of IgG1 production against M3R [129]. Purified IgG fractions from NOD.B10-*H2b* mice were capable of reducing saliva secretions in normal C57BL/6 mice as opposed to fractions isolated from sera of NOD.B10-H2b. Stat6^−/−^ mice that inhibited saliva flow rates when infused into naive C57BL/6 mice [129]. Thus, it is essential to note that IL-4, the primary cytokine produced by Th2 cells, plays a part in the isotype class switching to produce pathogenic IgG1 auto-antibodies highlighting the significance of the IL-4/Stat6 pathway.

#### 4.1.3. Th17 Cells

The role of Th17 cells, has been studied extensively in the past decade in the pathogenesis of SS [130]. Both IL-6 and transforming growth factor (TGF)-β are required to induce naive murine CD4^+^ T cells to develop into Th17 cells, which are characterized by the expression of retinoic acid receptor-related orphan receptor γ (RORγ)t. In humans, the differentiation of Th17 cells occurs by activation of T cell receptor (TCR) signaling in the presence of TGF-*β* and IL-6 or IL-21 stimulation [131]. Other critical cytokines that play a role in the progression of the disease include IL-22 and IL-23. IL-22 is derived primarily from natural killer cells, but it is also produced by Th17 cells, and it has been identified in the mSG tissue of pSS patients [74]. IL-23, while not required for differentiation of Th17 cells, is a cytokine that is necessary for their survival and maintenance [132]. Th17 cells produce IL-17A (referred to here as IL-17) and five other IL-17 members which have also been described that are termed as IL-17B, C, D, E (or IL-25), and F with conserved residues in the c-terminal region that form homodimers [133]. Local IL-17 protein production and mRNA levels, together with IL-6 and IL-23 mRNA, have been shown to increase with the progression of lesion severity in mSGs of pSS patients [134]. Th17 cells are the primary producers of IL-17A and IL17F and other cytokines such as TGFβ, IL-6, and IL-12, which have been detected in the plasma and saliva of pSS patients [134]. The nuclear receptor RORγt plays an indispensable role in the differentiation of Th17 cells as increased presence and activation indicates an increase in autoimmunity [135]. PBMCs from SS patients have the capacity to secrete IL-17 and IL-12 which skew naïve CD4^+^ T cells to Th1 and Th17 cells respectively, thereby facilitating initiation of the auto-immune cascade [136]. IL-21 expression in SGs has also been associated with hypergammaglobulinemia and patients with primary SS [137]. Th17 cells display the CD4^+^ CD161^+^ phenotype in circulation and have been found to be increased at advanced stages of the disease [138]. There are other subsets of marker specific T cells that contribute to disease progression. CD4^−^CD8^−^ double negative T cells are a subset that is capable of producing IL-17 and has been correlated with more severe glandular infiltration and is present during the formation of GCs [139]. Another direct set of Th17 cells that secrete IL-17 consistently in the periductal infiltrates of all mSGs, has been identified with the level of expression directly correlating with the severity of glandular inflammation and as a result destruction of healthy gland tissue [134].

Other Th17 cytokines that include IL-17 and IL-23 expression in SGs cause an increase in Tbet expression in the pre-disease phase in the C57BL/6.NOD-*Aec1Aec2* model [140]. The systemic effect of IL-17 on sexual dimorphism has been elucidated by genetically ablating IL-17 in C57BL/6.NOD-*Aec1Aec2* mice. It has been observed that the elimination of IL-17 reduces sialadenitis more drastically in females than in males [141]. The TCR repertoires of Th1 and Th17 cells in SG infiltrates have been found to be restricted, with an increase in the number of pathogenic effector T cells in the glands with a sex-based selection bias of TCR repertoires [142]. Furthermore, it has been observed that transferring Th17 cells in IL-17 deficient mice, restores the SS disease phenotype, highlighting the key role of Th17 cells in the inflammatory cascade and subsequent disease progression [143].

The function of RORγt overexpression in naive CD4^+^ T cells has been elucidated in RAG deficient mice showing the development of pSS phenotype upon transfer of RORγt-overexpressing CD4^+^T cells that induce sialadenitis. The findings in IL-17-deficient mice therefore, suggest that IL-17 is essential for the development of sialadenitis [144]. Gene therapy studied in the C57BL/6.NOD-*Aec1Aec2* mice has explored the role of cytokines like IL-27. Induction of IL-27, a natural inhibitory cytokine of Th17 expression, was found to down-regulate or reverse SS in C57BL/6.NOD-*Aec1Aec2* mice via a recombinant adeno-associated virus (rAAV) 2-IL27 vector injection. Th1 activation and inhibitory activity of Th17 cells was observed [145].

M3R-reactive CD3^+^ T cells play a pathogenic role in the development of murine autoimmune sialadenitis (MIS), which mimics SS [146]. M3R is the primary receptor subtype that promotes fluid secretion in salivary acinar cells. Both interferon IFN-γ and IL-17 are required for induction of SS in MIS, indicating that M3R-reactive Th1 and Th17 cells contribute to the pathogenesis of autoimmune sialadenitis. Thus, MIS is used to analyze the effectiveness of RORγt antagonists [147]. As mentioned, anti-M3R autoantibodies have been proposed to contribute to secretory dysfunction in SS. Iizuka et al. showed that transferring the M3R deficient splenocytes to RAG deficient mice lead to Th1 and Th17 infiltration in SGs and pSS like symptoms. Lymphocytic infiltration and destruction of epithelial cells in the SGs indicated that M3R reactive CD3^+^ T cells played a pathogenic role in the development of autoimmune sialadenitis [146].

In the lacrimal glands, the lymphocytic infiltration and the presence of IL-17 can also be observed. IL-17 conjunctival mRNA and protein expression in tears is observed to be higher in pSS as compared to non-SS patients exhibiting dry eye disease [148], whereas percentages of peripheral IL-17-producing CD4^+^ T cells are shown to be similar between pSS patients and controls. The importance of Th17 was further supported in animal models of SS. IL-2Rα (CD25) knockout mice develop autoimmunity and lymphoproliferative disorders and produce significantly higher levels of IL-6, TGF-β1, IL-23R, IL-17, IL-17F, IL-21, IL-10, and IFN-γ mRNA in the cornea and conjunctiva. This promotes autoimmune lacrimal-keratoconjunctivitis with symptoms closely resembling SS. Th-17 cells are shown to produce IL-17 that overlap with the peak severity of corneal epithelial disease [149]. A clinical trial using anti-IL-17 failed to improve dry eye in SS patients, which makes the role of Th17 cells in disease progression within LGs ambiguous [150].

#### 4.1.4. T Regulatory Cells (Tregs)

Tregs possess suppressive activity towards autoreactive lymphocytes via either cell-cell contact or the release of soluble mediators including IL-10 and TGF-*β*. The commitment of a naïve T lymphocyte towards a Treg phenotype is dependent on a specific cytokine microenvironment and of the expression of the forkhead box protein P3 (FoxP3) transcription factor [151]. Understanding the role of Treg cells in SS pathogenesis has been complicated by studies reporting mixed and controversial results. The inconsistencies in results can be explained at least in part by the different strategies employed to assess Treg cells in the course of disease progression. Studies follow two approaches of either enumerating the proportion of circulating Treg cells according to the high surface expression of CD25^high^ cells or combining surface expression with the co-expression of FoxP3, the most specific marker of Treg cells. An increase of circulating FoxP3^+^ cells in pSS biopsies correlates with worse clinical disease has been observed as shown by Sarigul et al., similar to FoxP3^+^ cells circulating in patients with RA [143,152,153]. Several studies report a reduction of peripheral blood Treg cells [78,154,155,156,157] and highlight an association between the reduction of these cells and exacerbated clinical symptoms. Szodoray et al. have proven that Treg cell reduction resulted in prevention of extra-glandular manifestations [78]. Contrary to these results, other groups report increased circulating Treg cells in pSS patients that show clinical symptoms, with no glandular manifestation and no serological features [152,158], and in a few cases CD4^+^CD25^high^ cell percentages are similar in the peripheral blood of pSS patients and controls [153,159,160].

Disease activity does not influence the number of circulating Treg cells and the disease presents as either being a mild stable polyclonal hypergammaglobulinemia, as was the case for one group (inactive) or a more severe polyclonal hypergammaglobulinemia (active) [160], as was the case for another group. Other murine studies on Tregs in SS include the treatment of TSP1-KO mice with TSP1-derived peptide to prove attenuation of the clinical symptoms of SS-associated dry eye in TSP-1 deficient mice. This demonstrates that an increase in Treg cells, which reduce Th17 cells, can attenuate disease symptoms. TGF-β plays a pivotal role in differentiation for immunosuppressive FoxP3^+^Tregs, where an increase is evident in biopsy specimens with mild and moderate inflammation which is disproportionate to escalating pro-inflammatory Th17 populations in advanced disease [134].

There is an increase in the frequency of CD4^+^Foxp3^+^ Tregs observed with age in the cervical lymph node (CLN), spleen, and LG of NOD.B10.H2b mice. These CD4^+^CD25^+^ cells lose suppressive ability, while maintaining expression of Foxp3 and producing IL-17 and IFN-γ. Furthermore, an increase of Foxp3^+^IL-17^+^ or Foxp3^+^IFN-γ^+^ cells was observed in the LG and LG-draining CLN of these mice [161]. The role of Tregs is uncertain because of a balance in between Tregs and Th17 cells [162]. Further soluble mediators, such as TGF-β, the level of which is increased in SGs of SS patients compared to controls, is required for both Treg and Th17 cell development [163,164].

#### 4.1.5. T Follicular Helper Cells (Tfh)

T follicular helper (Tfh) cells are specialized providers of T cell help to B cells, and are essential for GC formation, affinity maturation, and the development of high affinity antibodies and memory B cells. Tfh cell differentiation is a multi-factorial process involving B cell lymphoma 6 (Bcl6) and other transcription factors that are usually upregulated in autoimmunity [165]. B cell depletion therapy by Rituximab has been used in patients with pSS, where it decreased the elevated levels of circulating Tfh cells and improved the symptoms of patients, illustrating the crucial role of the crosstalk between B cells and Tfh cells in pSS [166]. Tfh cell differentiation is driven by the transcription factor Bcl-6 and activates Tfh cells to express high levels of Inducible T-cell costimulator (ICOS) and Programmed cell death protein 1 (PD-1) [167]. Tfh cells are important in the formation of GCs and primarily show presence of CD84 as a cell surface marker. CD84 ^+^ PD-1^+^Bcl6^+^ Tfh cells have been identified in organized structures with high focus scores and are in close proximity with Bcl6^+^ B cells, suggesting an association with increased disease severity in SS [168]. Tfh cells facilitate T cell–dependent B cell responses, mainly by secretion of IL-21, a primary driver of B cell activation and differentiation towards plasma cells. Increased frequencies of Tfh cells have been associated with several autoimmune diseases [169,170]. Cohorts of Tfh cells have been defined where the frequencies of circulating Tfh (cTfh) cells, defined as CD4^+^CD45RA^−^CXCR5^+^PD-1^+^cells, and are increased in pSS patients. Tfh cells within glandular tissue cannot be easily identified due to overlapping CXCR5 expression with B cells. Detection by immunohistochemistry and quantification of these cells by flow cytometry is difficult because biopsies are processed into cell suspensions using enzymatic digestion, and in the process CXCR5 expression is lost [171]. The function of CXCR5 positive Tfh cells is thus directly related to the secretion of IL-21 mediating B cell maturation, proliferation, and GC formation.

#### 4.1.6. Cytotoxic T Cells/ CD8^+^ T Lymphocytes (CTLs)

CTLs are best known for their destruction of virally infected and tumor cells. They produce the pro-inflammatory effector cytokines TNF-α or IFN-γ. The effector function of CD8^+^ T cells follows recognition by the T lymphocyte T-cell receptor of major histocompatibility complex class I (MHC I) molecules loaded with the relevant antigenic peptide, expressed at the surface of the target cells. Due to their lytic capacity, these cells represent key effectors in various autoimmune diseases [172]. Tissue resident memory CD8^+^ T cells act as mediators of SG damage in murine models of SS but the pathogenic significance of CD8^+^ T cells is unclear as limited studies have been performed to illuminate their role. CD8^+^ T cells have been observed within labial SGs infiltrates of patients with SS. They tend to colocalize with salivary duct epithelial cells and acinar cells, and they potentially produce pro-inflammatory cytokines. Infiltrating lymphocytes with a CD69^+^CD103^+/−^ tissue-resident phenotype and increased IFN-γ production were prominent in the submandibular glands of p40^−/−^CD25^−/−^ mice used as a murine model of SS, indicating initiation of the inflammatory pathway. This knockout mitigated symptoms and reduced progression of the disease, elucidating the role of CD8a in SS [173]. Subsequently, genetic ablation of IFNγ resulted in decreased CD8^+^ T cell infiltration and glandular tissue destruction. More importantly, depletion of CD8^+^ T cells fully protected mice against the pathologic manifestations of SS, even after the onset of disease [173]. A subset of these CD8^+^ T cells show an activated phenotype, as reflected in higher expression levels of HLA-DR where increased proportions of HLA-DR^+^ T cells are associated with higher disease severity [174]. Increased HLA-DR expression has been observed in both CD4^+^ and CD8^+^ T cells in the blood of patients that were positive for anti-SSA antibodies. The frequencies of HLA-DR-expressing activated CD4^+^ and CD8^+^ T cells in blood correlated with high ESSDAI scores [174]. Whole blood transcriptomic studies, serum proteomics, and peripheral immunophenotyping show a proportion of activated CD8^+^ T cells in blood that indicate an activated gene signature profile [175]. CXCR3 is necessary for the migration of CD8^+^ T cells into SGs [176]. High ESSDAI scores correspond to the activation of CD8^+^ T cells in lymphoid organs, CXCR3 upregulation, and consequent migration to the SGs [174]. Within the LGs and SGs of NOD mice, CD8 T cells proliferate, express an activated phenotype, and produced inflammatory cytokines. Transfer of purified CD8 T cells isolated from the cervical lymph nodes of NOD mice into NOD-severe combined immunodeficiency recipients resulted in inflammation of the LGs, but was not sufficient to cause inflammation of the SGs as observed in the study by Barr et al., demonstrating that CD8 T cells have a pathogenic role in LG autoimmunity [177].

Tissue auto-antigen responses and activated CD8^+^ T cells have not been well characterized in explaining autoimmune diseases like SS. Identifying human leukocyte antigen class I (HLA-I) binding peptide motifs gives insight to CD8^+^ T cells involved in pSS, but their role in pathogenicity and progression of the disease besides secretion of cytokines, primarily TNF-α, and IFN-γ is still unclear. New findings in the pathophysiology of CD8^+^ T cells in autoimmunity and a better understanding of their activation may provide opportunities for the development of targeted immunologic therapies in various autoimmune disorders.

### 4.2. B Cells

#### 4.2.1. Marginal Zone B Cells

Marginal zone (MZ) B cells are a class of innate-like lymphocytes positioned in the marginal zone of the spleen, inhabiting a junction between the circulation and lymphoid follicle [178,179,180]. While some differences exist between human and mouse MZ B development and function, in both organisms the positioning of MZ B cells within the spleen allows them to act as antibody generating first responders against pathogens in the blood [178,181]. While MZ B cells occupy a critical niche between innate and adaptive immunity, MZ B cells expansion has been associated with autoimmunity and previous studies have determined that MZ B cells possess polyreactive BCRs that can be potentially be self-reactive [178,182,183,184,185]. Among the most serious complications of SS is the increased incidence of B cell lymphoma [3]. Investigation into the types of non-Hodgkin Lymphoma (NHL) tumors within a cohort of 58 pSS patients identified that the two most commonly occurring types of tumors were indolent extranodal MZ B-cell lymphoma of the mucosal associated lymphoid tissue (MALT) at 59%, and nodal MZ lymphoma at 15%, indicating the importance MZ B cells within the disease [186]. MZ-like B cells have been found to be increased in the SGs and peripheral blood of pSS patients [187].

Investigations in SS mouse models have provided additional clues regarding the role of MZ B cells in the disease process. Mice transgenic (tg) for BAFF are known to develop autoimmune symptoms similar to SLE and SS [188,189]. In order to study the role of MZ B cells in this model, Fletcher et al. generated BAFF tg mice lacking lymphotoxin-β (LTβ), as mice lacking LTβ will fail to develop MZ B cells [190]. The authors discovered that while the mice still developed nephritis, they did not develop severe sialadenitis associated with the SS phenotype, suggesting a critical role played by MZ B cells in this aspect of disease [190]. A separate study investigated the role of MZ B cells within the IL-14α transgenic mouse model (IL14αtg) [191]. Elimination of MZ B cells within the IL14atg model restored saliva flow rate, removed lymphocytic infiltrations into the SGs, and prevented formation of autoantibodies [191]. Considering that depletion of MZ B cells improves disease in two separate SS mouse models, targeted depletion of MZ B cells has attracted attention as a potential therapy. Ly9 (CD229) is a cell surface receptor highly expressed on MZ B cells, and anti-Ly9 has been demonstrated to selectively target and deplete MZ B cells [192]. Treatment of NOD.H-2^h4^ SS model mouse with anti-Ly9 reduced both SG and renal infiltration, and also decreased ANAs and RF [193]. Considering the overwhelming involvement of MZ B cells in NHLs of SS patients, anti-Ly9 antibodies likely represent the first of many strategies to target MZ B cells for depletion in SS.

#### 4.2.2. Memory B Cells and Plasma B Cells

Memory B cells are the important cell type that is involved in SS pathogenesis due to their ability to maintain memory for a given antigen in the absence of constant antigen stimulation [194,195]. They are characterized by CD27^+^ expression and BCR somatic hypermutation [196,197]. SS shows an increase in accumulation of CD27^+^ memory B cells and plasma cells within the SGs infiltrate and the peripheral blood of patients. The glands show a distinct cytokine profile that includes adhesion molecules, cytokines, and B-cell chemokines CXCL13 and CXCL12 [88,90]. CXCL13 is the key cytokine responsible for the homing of B cells to the SGs. GC formation in the SGs is facilitated by CXCL13 secreted by Tfh and fDCs. Interaction with its corresponding receptor CXCR5 on B-cells regulates B-cell movement between different tissues. In the SGs, CXCL13 guides B cell entry into the follicles that causes lymphoid organization visualized in SG biopsies of SS [198,199,200]. CXCL13 over-expression in inflamed glands of patients with pSS plays the primary role in the recruitment of circulatory CXCR5 expressing CD27^+^ memory B cells, attracting the subpopulation of peripheral CD27^+^ memory B cells into the inflamed glands where they then reside [88,90].

Plasma B cells are terminally differentiated cells of the B lymphocyte lineage, the primary cell type that produces antibodies, and thus are drivers of antibody-mediated immunity [201]. They are maintained for extended periods, making them an essential component of immune memory and thus linking them closely to memory B cells from which they can differentiate [202]. BAFF, a member of the TNF family, is secreted by inflammatory cells and is needed for prolonged plasma cell survival and sustained Ig production by plasma B cells [203]. The glandular microenvironment is generally rich in BAFF, promoting accumulation of CD27^+^ B memory cells resulting in more IgG producing plasma cells in the tissue [203,204,205]. BAFF has a role in B cell maturation, class switching, survival, and proliferation especially in advanced disease and is produced by SGEC, DC, macrophages, activated T cells, and also B cells [204]. Mice transgenic for BAFF possess increased numbers of mature B and effector T cells, and develop high levels of circulating immune complexes, glandular Ig deposition, and anti-DNA antibodies [189]. The patients of pSS have auto-reactive plasma B cell infiltrates that produce anti-SSA/Ro or anti-SSB/La autoantibodies. These infiltrates appear from the differentiation of CD27^+^ memory B cells recruited from circulating blood and from B cells generated in ectopic SG GCs. Other notable cytokines include CXCR3 and CXCR4, whose expression on activated B cells leads to the migration of the plasma cells to the site of inflammation and causes the attraction of lymphocytic cells to the SGs [206,207,208,209,210,211].

B-cell-targeted mAbs (see below), largely rely on two mAb Fc-dependent mechanisms: antibody-dependent cellular cytotoxicity (ADCC) and complement-dependent cytotoxicity (CDC).

Rituximab, a mouse/human chimeric IgG1 mAb, was the first B-cell targeting therapeutic antibody approved by the US Food and Drug Administration [212,213]. Ocrelizumab a second generation CD20 mAb(rhumAb 2H7v.16) is a humanized CD20 mAb, which binds a different but overlapping epitope from rituximab [214]. Furthermore B-cell survival, differentiation, and functional properties are tightly regulated by a variety of cytokines and chemokines. Targeting survival and differentiation factors with specific mAbs or fusion proteins is an alternative approach to targeting B-cell surface antigens for active cell depletion [215].

## 5. Conclusions

**Figure 1:**
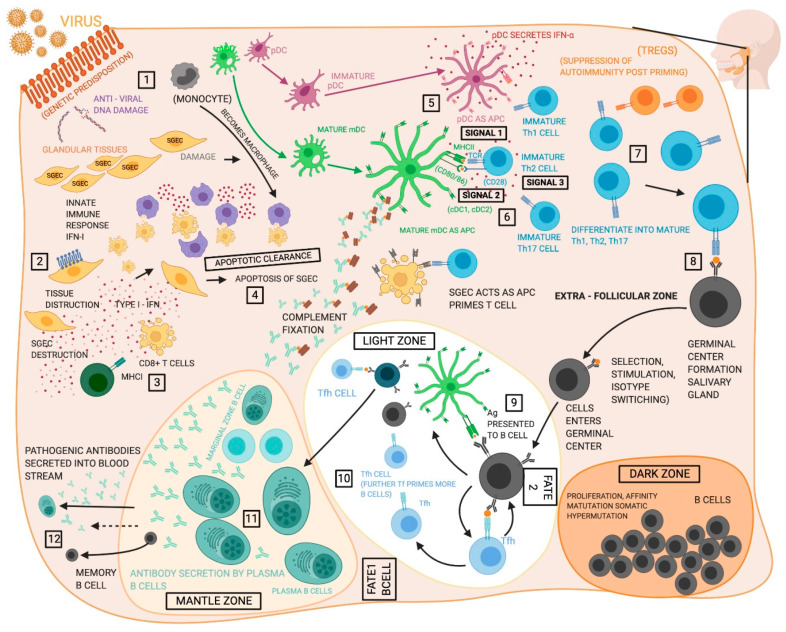
Proposed functions of different cell types in the pathogenesis of Sjögren’s syndrome (SS). (1) The initiating events in the development of SS remain unclear, but evidence suggests the disease proceeds following an environmental trigger on a susceptible genetic background, likely a viral infection. (2) Salivary gland epithelia cells (SGECs) experience increased apoptosis and act as sources of inflammatory cytokines and chemokines within the salivary gland (SG). (3) CD8^+^ T cells are poorly understood in SS, but may contribute to tissue destruction in the glands. (4) Macrophages participate in tissue destruction through the release of proteases and cytokines and display reduced efferocytosis allowing unremoved apoptotic cells to act as sources of self-antigen. (5) Type I interferon (T1-IFN) both initiates antiviral activity and exerts an activating effect on cells of the immune system. T1-IFN is produced by multiple cell types but is closely associated with plasmacytoid DCs (pDCs). (6) Myeloid dendritic cells (mDCs) are the dominant antigen presenting cells to T cells, however, macrophages and SGECs also participate. (7) Antigen presentation allows for activation of CD8^+^ T cells and the Th1, Th2, and Th17 CD4^+^ T cell subsets, which can then contribute to various aspects of the disease pathology. Th1 cells enter the glands and compose much of the early infiltrates and exacerbate the inflammatory environment with the production of type II interferon, while Th17 cells play an increasingly well recognized role in SS as sources of cytokines including IL-17. Conflicting evidence has caused the roles of regulatory T cells (Tregs) remain indistinct in SS. Th2 cells support the humoral autoimmune response through cytokines incusing IL-4. (8 and 9) T follicular helper (Tfh) cells support B cell development in germinal centers (GC) that include follicular dendritic cell (fDC) networks. Germinal centers exist in the spleen, but about 25% of SS patients develop ectopic germinal centers in the SG containing Tfh and fDC. B cells in the germinal center undergo proliferation, somatic hypermutation and affinity hypermutation in the dark zone of the germinal center. (10) B cells then proceed to germinal center selection by fDCs in the light zone and either leave the germinal center as memory B cells or antibody producing plasma cells (Fate 1), or return to the dark zone for further affinity maturation (Fate 2). (11) The MZ B cells function as part of the adaptive immune system carrying antigens to the germinal centers for more efficient generation of memory B cells and glandular MZB cells are proliferative, activated, and produce autoantibodies. Lastly, (12) plasma cells exhibit hyperactivity and are responsible for the production of pathogenic autoantibodies. Created with BioRender.com.

SS is a heterogeneous disease with a wide spectrum of severity. Both human and mouse studies of SS indicate an involvement of multiple cell types in producing local inflammation in pSS and SS-like disease as summarized in Table 1. The innate immune response is crucial for the pathogenesis of SS and is implicated in disease initiation, contributing to starting the immune cascade and guiding adaptive immune responses. The activation of innate immune cells is considered to be indicative of disease onset while T and B cell infiltrates indicate driving responses in more severe cases that is reflected in glandular tissue destruction. Both cellular and cytokine repertoires that mediate the skewing, activation, and differentiation of different cell types in peripheral blood and exocrine tissue initiate the epithelial cell activation and in turn the innate immune response as illustrated in Figure 1. This process ultimately leads to chronic autoimmune responses resulting from adaptive immune cells. To date, numerous studies have been performed to identify the cells relevant to autoimmune disease and to understand the individual contributions of all interacting cell types. As a result, comprehending contributions of individual cell types and their interactions is of great importance for elucidating disease pathogenesis and the development of effective therapeutic interventions.

## Figures and Tables

**Table 1:** Immune cells and their functions involved in SS.

Cell Type	Immunity	Function	References
Dendritic cells	Innate	• mDC are increased in pSS SGs, pSS patient mDCs have increased IL-12p40 secretion and HLA-DR expression. • pDC identified in pSS SGs, pSS patient pDCs are decreased in circulation but show increased activation. • fDC can be organized into fDC networks within functional ectopic GC in the SGs.	[41,42,44]
Macrophages	Innate	• Macrophage infiltration correlates with disease severity in pSS. • Infiltrating macrophages express IL-18 and proteases allowing them to contribute to inflammation and tissue destruction. • pSS monocytes and SS mouse model macrophages display impaired efferocytosis.	[38,59,61]
Salivary gland epithelial cells (SGECs)	Innate	• SGECs can operate as non-professional APCs and as sources of multiple inflammatory cytokines. • SGECs are sensitive to TLR induced apoptosis.	[86,101]
Th1 cells	Adaptive	• Play a role in the organ development of SGs. They prevent normal acinar cell proliferation and maturation. • Secrete IFN-γ that induces expression of glandular adhesion molecules allow the influx of inflammatory cells into SGs. • In-vitro exposure of acinar cells to IFN-γ causes alterations in tight junction components as observed in the SGs of patients with pSS.	[97,108,109,112]
Th2 cells	Adaptive	• Secrete IL-4 that prevents secretory function. • Secretion of IL-4 causes formation of IgG1 isotypic autoantibodies against M3R indicating a critical role of IgG1, IgG2a, IgG2b, IgG3, IgM, and IgA isotype switching in SS. • *Stat6* gene also prevents IgG1 production against M3R and also plays a part in the isotype class switching.	[108,129,216]
Th17 cells	Adaptive	• They are stimulated by cytokines that play a role in the progression of the disease such as IL-22 and IL-23. • IL-22 is derived primarily from natural killer cells, is produced by Th17 cells, and it has been identified in the mSG tissue of pSS patients. • Th17 cells produce IL-17A (refer to as IL-17) and five other IL-17 members which have also been described that are termed as IL-17B, C, D, E (or IL-25), and F with conserved residues in the c-terminal region that form homodimers. • Local IL-17 protein production and mRNA levels, together with IL-6 and IL-23 mRNA, have been shown to increase with the progression of lesion severity in mSGs of pSS patients. • Conjunctival RORγT mRNA and protein expression in tears is observed to be higher in pSS as compared to non-SS patients exhibiting dry eye disease. • IL-21 expression in SGs has also been associated with hypergammaglobulinemia and patients with primary SS.	[133,134,152,155,163,217]
T regulatory cells (Tregs)	Adaptive	• Important for the induction and maintenance of peripheral tolerance therefore, they are key in preventing excessive immune responses in SS. • Suppressive activity towards autoreactive lymphocytes via either cell-cell contact or the release of soluble mediators that notably include IL-10 and TGF-*β*. • Reduction of peripheral blood Treg cells in humans that lead to exacerbated clinical symptoms of SS. Role of Tregs is uncertain because of a balance in between Tregs and Th17 cells.	[152,154,218,219]
T follicular helper cells (Tfh)	Adaptive	• Specialized providers of T cell help to B cells, marked increase of Bcl6 and other transcription factors that are usually upregulated in SS. • Important in the formation of GCs and primarily show presence of CD84 a cell surface marker, observed in SS. • The function of CXCR5 positive Tfh cells is directly related to the secretion of IL-21 mediating B cell maturation, proliferation, and GC formation.	[165,166]
Cytotoxic T cells/ CD8^+^ T cells (CTLs)	Adaptive	• They produce the pro-inflammatory effector cytokines TNF-α or IFN-γ. • Tissue resident memory CD8^+^ T cells act as mediators of SG damage in murine models of SS but the pathogenic significance of CD8^+^ T cells is unclear as limited studies have been performed to illuminate their role. • Tend to colocalize with salivary duct epithelial cells and acinar cells, and produce pro-inflammatory cytokines.	[172]
Marginal Zone B cells	Adaptive	• Stimulated by BAFF. • MZ B cells within the IL14atg model drive reduced saliva flow rate, lymphocytic infiltrations into the SGs, and formation of autoantibodies. • Possess self-reactive BCRs that cause complications of SS and the increased incidence of B cell lymphoma.	[174,179,189,192,193]
Memory B cells	Adaptive	• Maintain memory for SS antigens in the absence of constant antigen stimulation. • CXCL13 is the key cytokine responsible for the homing of B cells to the SGs. • CD27^+^ memory B cells, attract the subpopulation of peripheral CD27^+^ memory B cells into the inflamed glands where they reside and cause inflammation. • The primary cell type that produces antibodies, and thus are drivers of antibody-mediated immunity. • BAFF primary cytokine produced, that has a role in B cell maturation, class switching, survival, and proliferation especially in advanced disease and is produced by SGEC, DC, macrophages, activated T cells, and also B cells • Cause the formation of GCs in SGs and work antagonistically to Tfr cells.	[88,167,194,195,220]
Plasma B cells	Adaptive	• B cells that produce SS auto-antibodies with specific BCRs against auto-antigens after differentiation from Memory B cells or circulating peripheral B cells.	[189,203,221]
